# Becoming the best mom that I can: women's experiences of managing depression during pregnancy – a qualitative study

**DOI:** 10.1186/1472-6874-7-13

**Published:** 2007-09-11

**Authors:** Heather A Bennett, Heather S Boon, Sarah E Romans, Paul Grootendorst

**Affiliations:** 1 Leslie Dan Faculty of Pharmacy, University of Toronto, 144 College Street, Toronto, ON, M5S 3M2, Canada; 2 Department of Psychiatry, Faculty of Medicine, University of Toronto, Toronto, ON, Canada; 3 Centre for Research in Women's Health, Toronto, ON, Canada; 4 Department of Economics, McMaster University, Hamilton, ON, Canada

## Abstract

**Background:**

The purpose of this constructivist grounded theory study was to develop a theoretical model that explains women's processes of managing diagnosed depression when pregnant.

**Methods:**

We explored the experiences of 19 women in Ontario who were diagnosed with depression during their pregnancy.

**Results:**

The model that emerged from the analysis was becoming the best mom that I can. Becoming the best mom that I can explains the complex process of the women's journey as they travel from the depths of despair, where the depression is perceived to threaten their pregnancy and their ability to care for the coming baby, to arrive at knowing the self and being in a better place. In order to reground the self and regain control of their lives, the women had to recognize the problem, overcome shame and embarrassment, identify an understanding healthcare provider, and consider the consequences of the depression and its management. When confronting and confining the threat of depression, the women employed strategies of overcoming barriers, gaining knowledge, and taking control. As a result of counseling, medication, or a combination of both, women felt that they had arrived at a better place.

**Conclusion:**

For many women, the idea that depression could occur during pregnancy was antithetical to their vision of the pregnant self. The challenge for a pregnant woman who is diagnosed with depression, is that effective care for her may jeopardize her baby's future health. This provides a dilemma for about-to-be parents and their healthcare providers. Improved awareness of depression during pregnancy on the part of healthcare professionals is needed to improve the women's understanding of this disorder and their ability to recognize and seek help with depression should it occur during the prenatal period. Further qualitative research is needed to determine the specific aspects that need to be addressed in such classes.

## Background

Major depressive disorder (MDD) is a chronic, recurrent illness associated with considerable disability, impaired quality of life [1], and high economic costs [2]. It is common in most countries, with annual rates in the adult population ranging from 7% to 13% [3]. Depression is twice as prevalent in women as in men, [4,5] and has been identified as a leading cause of disease burden for women aged 15 to 44 years globally [6].

The mean age of onset of depression for females ranges from the early 20s to early 30s [3], coinciding with the childbearing years of a woman's life[4] The notion that pregnancy is a time of joyful expectation, a satisfying and fulfilling experience for all women, has been exposed as a myth [7,8]. It is now clear that some women develop depression during pregnancy, [9] while others with a history of depression are at risk for its recurrence[10] Indeed, an estimated 13% of pregnant women experience this disorder [11].

Most observers consider neurobiological and environmental factors, genetic predisposition, and acute and chronic stress to be implicated in the development of MDD [12-17]. The quality of interpersonal relationships, stress, locus of control, and psychiatric history are thought to have a direct effect on depressive symptom levels in pregnant women [18], while biological variables are thought to act indirectly through their effect on vulnerability to psychosocial stressors [19] To date, however, there has been no definitive analysis that adequately explains the onset, chronicity, severity, and relapse of depression in either the obstetric or postpartum periods [20].

Despite the burgeoning quantitative literature on the clinical outcomes of maternal depression (see Bennett et al [21], for an overview), little is known about pregnant women's subjective experiences of depression. A review of the literature failed to reveal any publications exploring women's experiences, feelings and perceptions of depression during pregnancy. This is surprising given recent findings that MDD is a leading cause of disease burden for women [6], the extensive exploration of women's experiences of postpartum depression (PPD) [22], and the current emphasis on disability due to mental health.

Given the intense concerns of women and their healthcare providers over possible teratogenicity and the behavioural impact of antidepressant use on the infant, the treatment experience of depression during pregnancy is likely to differ substantively from that of PPD. Furthermore, it has been suggested that there may be a "different biologic basis or vulnerability" to depression in pregnancy than in the postpartum resulting in different symptom profiles [23]. Although existing qualitative literature exploring PPD may have some relevance to the experience of depression in the prenatal period, there may also be differences between the two reproductive phases and extrapolation from one to the other is risky.

The aim of this research was to explore women's experiences of depression during pregnancy and to develop a theoretical model for the processes they used to manage their depression. It is concerned with the women's personal experiences, how they make sense of those experiences, and how those meanings relate to their management of the disorder. Understanding how the women view this disorder and its management has the potential to provide fresh perspectives for the delivery of care.

## Methods

The methodological approach for this study was constructivist grounded theory [24]. Constructivist grounded theory, in contrast to both the original theory as conceived by Glaser and Strauss [25] and Strauss and Corbin's [26] modified version, emphasizes the constructivist element of the development of an interactive relationship and mutual construction of knowledge between researcher and participants [24,27,28]. Benoliel [29] suggests that this approach is appropriate when the phenomenon of interest involves understanding experiences, meanings, and motivations of everyday life and the basic processes people use to deal with social situations to which they must adapt. This is particularly the case when the goal is to "uncover the nature" of experiences with a phenomenon like illness [26].

## Conceptual framework

The conceptualization and design of this study was guided by a social constructivist paradigm [30] and a symbolic interactionist framework [31]. From the viewpoint of social constructivism, health and illness can be seen as socially constructed events [32,33]. A social constructivist approach is particularly well suited to depression research because depression often deals with an individual's construction of the world and their relationships within that world [34].

To understand the process of managing prenatal depression there must be an appreciation of the women's experiences, comprehension of their understandings of the world in which they live, and an understanding of their moral judgments [31,34]. Symbolic interactionists believe 1) that objects have meaning only through people's interactions with them in the environment, 2) that the meanings people have for things develops through social interaction, and 3) that those meanings are handled and modified by a constant and ongoing interpretive process by individuals [31]. Thus, symbolic interactionism provides a useful framework within which to study depression, a disorder "deeply connected with individual and collective interpretations" (p. 337) [35]. Furthermore, the fundamental assumptions of symbolic interactionism about the nature of self and the concept of change provide a structure within which the social interactions of the women, roles, emotions, and presentation of self can be examined in great detail [36].

## Ethics and recruitment

Permission to conduct the study was received from the relevant hospital and university ethics boards. Recruitment was conducted through a reproductive mental health program located in a major urban centre in southern Ontario. Purposively recruiting women from the reproductive mental health program and including only women with a psychiatrist-diagnosed depression maximized the opportunity of interviewing women who could provide the best and clearest examples of the phenomenon of interest [37].

Recruitment was a two-step process. First, healthcare providers at the program contacted potential participants by letter alerting them to the study and asking those interested in participating to telephone the first author (HAB). Second, women who contacted HAB were provided an explanation of the study, invited to ask questions, and an appointment was made to meet with women who wished to participate. In this way, the women could be reassured that their healthcare provider would not know of their decision regarding participation in this study. Potential participants were provided with a verbal explanation of the study and given the opportunity to ask questions during the initial telephone contact.

Women who agreed to participate were given as much time as they required at the beginning of the meeting to read the study information sheet/informed consent form, to ask questions, and satisfy themselves as to the conditions and implications associated with their participation in this study. Informed consent was obtained in writing prior to the start of the interview. Confidentiality was maintained by assigning each participant a code number. No participant identifiers were recorded on any source documents or transcribed interviews.

Of forty seven women who were notified about the study, twenty five contacted the researcher. No attempt was made to determine why the other 22 women did not contact the researcher. Two women did not fulfill inclusion/exclusion criteria because they had not experienced depression until the postpartum period. Two women who had agreed to be interviewed changed their minds prior to their interviews. Thus, 21 women were eligible and available to participate in the study. During the interviews it became apparent that one woman had not experienced depression until the postpartum period, and another had experienced bipolar not unipolar depression during her pregnancy. The interview data from those two women were excluded from the analysis. Therefore, nineteen women who had experienced depression during pregnancy, as diagnosed by a psychiatrist, participated in the study.

## Data collection

Data about the woman's experiences of depression were collected through individual, in-depth, semi-structured, audio-taped interviews. Women were invited to propose the time and location of the meeting so that they would feel more empowered in the interaction encouraging them to share their experiences [38,39]. Sixteen women elected to be interviewed in their own homes, the remaining three women were interviewed in a private room at the hospital.

All interviews were conducted by HAB and lasted between one and a half and two hours. Open-ended questions pertaining to the women's experiences associated with depression during pregnancy were posed and demographic data were collected. Each interview began with the question "Can you tell me what it was like for you being depressed while you were pregnant". This broad question served to "break the ice" (p. 660) [38] leading to conversation that provided information on the woman's views about depression, labeling, stigma, incidents contributing to her depression, her symptoms, the influence of depression on her relationships, her coping strategies, her experiences of help-seeking, and her feelings and beliefs about counseling and the use of antidepressants. While the goal of the study was to obtain information in all areas, individual experiences of each woman dictated how much time was spent discussing each topic. Questions were asked to validate the women's experiences and encourage them to show how they had reached particular conclusions [27]. The final question was "Is there anything that we haven't talked about that you would like to tell me or that you think I should know?" In this way, knowledge was mutually interpreted and co-constructed between the women and the interviewer in line with the principles of the constructivist paradigm [27]. Table 1 provides the core and probe questions used in the interviews.

**Table 1:** Interview guide – core and probe questions, asked in semi-structured interviews, that pertain to the women's depression experiences

**Can you tell me what it was like for you being depressed while you were pregnant?**
• How did the depression affect your everyday life?
• What affect did it have on your relationships with your partner, children, family, friends or work colleagues?
• How did you make sense of the symptoms that you were experiencing?
• How would you describe the process of becoming aware that what you were feeling may have been depression?
**Where there any events that you think contributed to your depressed mood?**
• Did you experience any other issues like troubles with your job, money, or with family or friends at that time?
• How did those events contribute to your depression?
**Before you went to see the doctor, what did you do about your mood and how you were feeling?**
• Did you seek help or support from anyone? Was the support provided by that person helpful?
• What else did you do to cope with your mood? Did anyone comment on the way you were coping?
• What do you think would have helped you cope with your depression?
**How did you make the decision to seek professional help for your mood?**
• Did you have any concerns about going to your doctor about your mood?
• What were your expectations of the doctor?
**What type of help did your doctor suggest for your depression?**
• Did you take medication or have counselling for your mood while you were pregnant?
• How did you make that decision about that? Who or what information helped you make that decision?
• How hard was that decision?
• How did you feel about the way that your doctor helped you to manage/treat your mood?
• Did you wish that your doctor had managed/treated your depression differently?
**How did you feel when you got the diagnosis of depression?**
• What did that mean to you?
• Did you tell anyone? Who? Why/why not?
• How did you cope with this 'label'?
• How did you change when you got the diagnosis of depression?
**How has your mood been since the birth of your baby?**
• How did your mood affect your attachment with your baby?
• How did your mood affect your sense of yourself as a mother?
**Is there anything else that you would like to tell me or that I should know?**

## Participants

The 19 participants were between 25 and 47 years of age (average age = 36), of varied ethnic backgrounds, and of relatively high socioeconomic status (SES) compared with the general Canadian population. Twelve were university educated, 7 had completed college, and the majority had an annual household income in excess of $75,000 (CDN). Nine women had one child at the time of the interview, nine had two children and one had three children (two women had twins). The pregnancy of interest, that is the pregnancy during which the woman was depressed, occurred on average 1.0 years (range = 0 to 2.5 years) prior to the interview. This relatively short time period between the pregnancy and the interview served to decrease the likely hood of recall error.

Only one woman was not living with the biological father of her baby. Seventeen sought mental health care during pregnancy and two delayed seeking care until the postpartum period. Fifteen women reported that they had experienced at least one episode of depression during their lifetime. Eight women were taking antidepressants prior to conception; three discontinued medication when planning their pregnancy and two upon confirmation of pregnancy. Four of the five who discontinued resumed antidepressants during pregnancy, the fifth resumed in the postpartum.

## Analysis

Grounded theory principles as developed by Glaser and Strauss[25], and refined by Strauss and Corbin [26], and Charmaz [24] were used to analyse interview data. As a method of theory development, constructivist grounded theory provides components for the systematic synthesis of social processes and for deriving theories through inductive analysis of empirical data collected in natural settings [24]. This method of analysis provides a set of systematic procedures for identifying categories and relationships between categories which arise in the data. It promotes description and explanation of the phenomenon under study and development of theory that is grounded in the data.

To gain an awareness of the experiences described by the women, taped interviews were listened to immediately following each interview. The first four interviews were transcribed verbatim by HAB and the remainder by a professional medical transcriptionist. HAB verified, and corrected where necessary, all transcripts against the recordings.

Each transcript was read multiple times to gain familiarity with its content, to identify conceptual categories within the interview data, and to examine relationships between those categories. Categories and relationships were verified against the data and patterns within the data were compared. The core category was identified, related to all other categories and validated against the data. The coding process, writing of memos, development of themes and links, and use of constant comparative analysis allowed the interview data to be "broken down, conceptualized, and put back together in new ways" [26]. Coding for the first five interviews was conducted by three members of the research team who met until consensus was reached regarding the coding scheme. HAB then coded the remainder of the interviews under the supervision of the second and third authors. Again, consensus was achieved, through regular meetings and coding sessions. Thus, peer review was incorporated via regular consultation between the authors. This provided the opportunity for discussion of issues and problems as they occurred. Feedback from those consultations influenced the ongoing coding, data analysis and emerging theory.

## Credibility of results

Constructivists establish relationships with the participants [24] enabling the co-construction of knowledge between the participants and the researcher [30]. The resulting constructions cannot be separate from those who make the constructions[28] and there can be multiple interpretations of the same data all of which are potentially meaningful [30,40]. In other words, the researcher is the primary research instrument, and as such, may have influenced the collection, selection, and interpretation of the data [28,41-43].

To address those concerns, the positionality of the researcher and confirmability of the results was through ongoing consultations between members of the research team and the creation of an audit trail to document the study process and procedures [28,41,43]. To ensure credibility study results were provided to several participants (those who were available and willing) for verification [44,45].

## Results

Women in this study accounted for their experiences of depression in terms of symptoms, the events and circumstances that they perceived contributed to their depression, and the steps that they took to address their depression. They told of an inability to function, an overwhelming anxiety, an inability to organize their thoughts, and trouble making decisions: tasks that "normally seemed easy, seemed huge" so that "getting through the day was horrendous". Overwhelmed and exhausted, the women confront their depression, and in the circumstance of their pregnancy, the depression was conceived as threatening to themselves, to their developing baby, and to their ability to mother the coming baby.

### Overview of Becoming the best mom that I can

Grounded theorists aim to uncover the social and psychological processes individuals employ to understand or address a basic social problem [46]. The problems confronting women in this study were a loss of control, an altered perception of self, and doubts about their maternal ability. The basic process for these women was becoming the best mom that I can. From the women's perspective, becoming the best mom that I can was the process of "doing everything I could" to implement control over the perceived threat to their pregnancy and their ability to care for the baby after birth. What the women do to regain control and reground the self, and how they do it, is juxtaposed with what they perceive to be the optimum situation for the developing fetus and 'soon-to-be' baby. They search relentlessly to find the most acceptable answer; they reflect upon the self, acknowledge the problem, and embark upon a journey to "put things into place before I have the baby".

**Figure 1:**
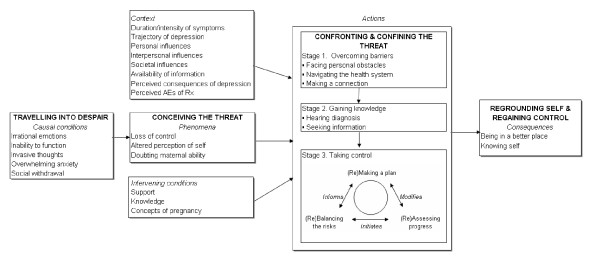
*Becoming the best mom that I can*: Theoretical model of managing depression during pregnancy.

This process consists of four major categories: Traveling into despair (causal conditions), Conceiving the threat (phenomena), Confronting and confining the threat (actions), and Regrounding self and regaining control (consequences). The full context in which *Becoming the best mom that I can *occurs will be discussed in greater detail elsewhere. Each major category is composed of subcategories as shown in Figure 1.

## Causal conditions of phenomena – Traveling into despair

The conceptual category of *traveling into despair *describes the women's experiences as symptoms of depression invaded their lives. Symptoms occurred at different stages of pregnancy for each woman; however, all described arriving at a point where they could not envisage themselves being any lower. They used phrases such as: "traveling down into blackness", "downward spiral", "wandering around in a blur", "constant state of despair," "downhill", "teetering", "going crazy", "falling apart", "crash and burn" "sliding into a depression", "into the pit of hell", and "over the edge".

The five properties that comprised traveling into despair were: Irrational emotions, Inability to function, Invasive thoughts, Pervasive anxiety, and Social withdrawal.

### Irrational emotions

Women spoke of frequent episodes of sadness, anxiety, irritability, anger, crying, and worry which led to feelings of "low self-worth, low self-esteem" and guilt. They were at a loss to understand or to explain their emotions which seemed to occur for no apparent reason and often without warning. One woman reported "just tears so close to the surface that anything would, I would just fall apart, and not just, it's not that sad, but it's almost irrational." (#7)

### Inability to function

The inability to function invaded every aspect of their lives. From the simplest of activities such as personal hygiene to the more complex tasks required in the performance of the work-day role, inside and outside the home, the women told of impaired performance. One woman, who had an older child, told of her inability to care for her son, "I felt bad because I wasn't at a point where I could take care of him how I usually take care of him." (#3)

### Invasive thoughts

Some women spoke of being unable to imagine their baby or imagining the baby "as this thing growing inside of me". A number of women had what they described as invasive thoughts:

Like, I had a thought of a woman stabbing me in my stomach when I was pregnant, you know. A woman that I saw in the elevator one day, you know, so, those weird passive thoughts. (#16)

Many women spoke of the fear of losing their mind or going crazy, and, while none attempted suicide, some had thoughts of suicide due to the intense pain their feelings were causing:

I wasn't planning to kill myself, or I hadn't thought of ways to do it, but I just wished every single day that I was dead, you know, I didn't want to live. (#21)

### Pervasive anxiety

Many of the women were anxious; they felt that something bad might happen but were at a loss to explain this feeling.

It's hard to explain this anxiety, because it's like something horrible is wrong, like something horrible happened to you. But nothing horrible happened to me. I don't know how to explain it. The anxiety was always there. (#15)

### Social withdrawal

Women withdrew from their world as they had known it. They did not maintain old friendships, nor did they establish new connections. Instead, they retreated into their homes which one woman described as becoming a "hermit". For some, interaction with others served only to highlight the intensity of their misery. Perhaps most illuminating was the following statement:

Even going to the [baby] showers is withdrawal, you know, balloons, and happy, and presents and friends, and nothing but people wanting to smile at you and you're just standing there like miserable, for no reason you can put your finger on. (#20)

## Phenomena resulting from traveling into despair – Conceiving the threat

This category represents the phenomena that result from the conditions encountered in traveling into despair. It occurs when the women try to make sense of their feelings and are unable to do so. Their ability to understand what is happening wanes as the symptoms of depression continue unabated.

Initially, the women "denied a lot of the stuff, kept it in, denied it, deluded myself and just kept going." They attributed their emotional state to being "too busy at work" or "I'm just pregnant, nothing is wrong". The women described their efforts to maintain a sense of normalcy in their lives as "putting on a happy face". By denying or ignoring what they were feeling they were able, at least for the short term, to pretend to themselves and to those around them that everything was on track. "So, that's what I do, I wear a mask for people, so be it."

As the women failed to improve they attempted to alleviate the way they felt by meditating, tuning out, and turning to comfort food. One woman, who found relief by watching television said, "It would be the only way that I could tune my brain out, stop the thoughts, you know, thinking". However, none found the relief for which they were searching. Despite their best efforts their coping strategies were not adequate. This was explained as, "Normally you can kind of problem solve your way out of it, but there was just no problem solving".

They acknowledged "that there was something wrong", that they "can't do it anymore" and they saw themselves as needing help. The feared that the depression was "a risk for the baby more than it is for me", they made the decision that they will "deal with this, and do whatever I have to do" to be "better before this baby comes." All women described an urgency that they attend to their depression prior to the birth of their baby.

The core categories of the subjective phenomena are; Loss of control, Altered perception of self, and Doubting maternal ability.

### Loss of control

A frightening loss of control permeated every aspect of the women's lives. They were unable to control their feelings, emotions, thoughts, and actions. The practicalities of the everyday were seen to be outside of their control; they had difficulty imagining how they would cope throughout the pregnancy and after the baby was born.

It was really frightening. I felt out of control. The anxiety that that provoked for me was just like right over the top. I thought how am I ever going to function, how can I possibly get through the entire pregnancy like this. (#18)

### Altered perception of self

The women looked to the familiar to try to come to grips with how they were feeling. They compared the current self with the previous self, with previous pregnancies and with others who were pregnant.

You see other pregnant women are jogging, buying baby clothes, working up until the last possible moment. You think why aren't I doing that. (#20)

For all women the current self was in conflict with their image of the pregnant self; many no longer recognized the current self as the old self.

I think that's initially what got me down the path, was just thinking this isn't like me. (#20)

### Doubting maternal ability

Many questioned their ability to mother, their decision to become pregnant in the first place, their ability to continue with the pregnancy, and they lacked hope for the future.

I just questioned a lot of like what am I doing, why am I pregnant, why did I try to get pregnant. (#16)

In addition, many women believed that their antenatal depression would harm their developing baby; they feared that they would develop PPD, and that they would be unable to care for their family and the coming baby.

I just had lots of fears that I wasn't going to be able to be a mom, because I sometimes felt so disabled. (#24)

## Context in which the strategies of becoming the best mom that I can developed

The strategies employed by the women in becoming the best mom that I can developed within the following interacting contexts: duration and intensity of depressive symptoms, perceived consequences of depression and of antidepressant use, personal, interpersonal, and societal influences, and availability of information. These contexts, which were influenced by both the causal conditions and the resultant phenomena, were paradoxical and interacted to create a tension that intensified the women's uncertainty about their strategies.

The women saw themselves as "getting worse", when they had "more bad days than good". Many described a depression so intense that it "affected every minute of the day" and that they "couldn't see it ending". They compared how they were feeling to their expectations of pregnancy which were shaped by past experiences and from what they had read in "pregnancy books, parenting magazines, and talking to others who were pregnant".

Women who had been pregnant previously and had not experienced depression recognized their mood as inconsistent with their previous pregnancy. It was that knowledge that helped them to determine their need for professional help. Women who had experienced depression and its management prior to pregnancy had the knowledge that enabled them to assess their level of depression. They recognized when they needed to seek professional help and they had intimate knowledge of the effect of counseling and antidepressant use on their mood. For women who were expecting their first baby and had not been depressed prior to pregnancy the decision was based upon their expectations. Many imagined the pregnant self would be "all roses and just so excited and everything".

I think that I had this expectation that when you are pregnant everything is rosy and perfect, and you are aglow. All these things we're sort of led to believe. So, I think when reality hit, I felt like crap. (#13)

On the other hand, personal, interpersonal, and societal influences, and availability of information acted as obstacles to their actions. Some women were embarrassed to seek care, others experienced difficulties forming therapeutic alliances with healthcare providers. Some women were fiercely "against medications" and others feared that they would be judged to be a bad mother if they took antidepressants while pregnant. When information provided by physicians and psychiatrists conflicted, women were confused and uncertain as to their course of action.

## Intervening condition that influenced becoming the best mom that I can

The condition that intervened and influenced becoming the best mom that I can was the type and degree of support that women perceived to be available to them. Most women in this study were in stable marital relationships and described their husbands as providing (in the main) practical and emotional support. Their husbands accompanied them, on at least one occasion, to the psychiatrist, and participated in discussions about treatment strategies. However, being seen by their husbands as "not having a grip on things" was upsetting and embarrassing for the women. Some women were not entirely convinced that their husbands, as supportive as they were, were totally aware of the depth of their emotional problems.

He boosts me up, but at the same time, sometimes I feel like he's really missing the point sometimes, or do you really know that I am really struggling here to keep it together.

Three women perceived their partners to be unsupportive. Those women actively excluded their partners from participation in, or discussion of, the management and treatment of their depression.

Few women described seeking support from parents or siblings. The majority delayed telling their families about their depression until after they had sought professional care; some women did not want to be a "burden", others were "ashamed". A number of women, who described their parents as being very ill or their families of origin as "dysfunctional", delayed, avoided, or simply were unable to discuss their concerns.

None of the women sought support from friends. Many were embarrassed about how they were feeling, convinced that they would not be understood, and feared that they would be "judged":

I mean it felt like a failure to me. I felt if I told my friends, I felt like I might be judged by it.

We're pretty judgmental about mothers; I mean mothers are the worse culprits, mothers judging other mothers. Then non-mothers judging non-mothers, and sometimes I think women are harder on each other than men are.

Unable to talk to others, the women's sense of loneliness and their sense of "being the only one" was intensified.

## Strategies for becoming the best mom that I can – Confronting and confining the threat

In the presence of the contextual and the intervening conditions, the women began the process of *confronting and confining the threat*. For the women in this study, that process involved seeking care from a psychiatrist experienced in the field of reproductive mental health and consisted of three stages: Overcoming barriers, Gaining knowledge, and Taking control. Each stage was composed of several properties as shown in Figure 1.

### Stage 1. Overcoming barriers

#### Facing personal obstacles

Women who had not experienced depression prior to pregnancy struggled to understand what was happening and challenged themselves saying, "there is no reason – there is no physical reason why I couldn't do more". (#20) They were ashamed and concerned that they would be viewed by others as being "incredibly lazy", or "wanting attention", and that what they were feeling would not be seen as "real". The act of asking for help was seen to be an admission of failure:

So, just swallowing my pride and going outside of the home, was a big step. Once I committed to saying that I have to tell someone, it was easier. (#16)

For some women, feelings of shame continued unabated throughout the period of their treatment. One woman explained how she felt when attending her scheduled appointments with the psychiatrist as:

Even when I was going to the psychiatrist in the hospital, I was worried that I would run into somebody. (#10)

#### Navigating the healthcare system

Having committed to telling someone the women approached their obstetric care provider or their family physician for help. For many, this resulted in being referred directly to a mental healthcare professional. One woman, who had been under the care of a midwife, said:

They [midwives] were fantastic. I don't know what I would have done, really and honestly had I not had them noticing stuff and asking me questions, because I was really, really withdrawn,. They actually gave me a referral and all of that. (#4)

Others encountered obstacles as they endeavored to navigate the health care system. Obstetricians were regularly considered to be "too busy" or to have "zero interest in me as a person". Women perceived that "there really wasn't time to talk" with their obstetricians about their emotional health and that they were considered as "purely a body". One woman, who had a particularly difficult time finding professional help said:

I guess she didn't have time for the counseling or to look into it, to talk to me further about it. She made it clear from the beginning that if you had any other medical problems other than pregnancy, that you would have to see your other doctor. (#15)

#### Making a connection

Making a connection with "the right" mental health professional was not immediate for all women. Because of their fear of not being believed, they needed to speak to someone who would understand and not judge. They needed "somebody who specializes in this area", someone who "recognized the complexity of my life as a woman and a mother", that they felt was "asking me the right questions", who was "understanding, sympathetic" and one who had "the time to listen". One woman "instantly felt better" because there was a good connection with her psychiatrist. Some women felt more comfortable when they were able "to speak to a woman". As one woman stated:

Someone that I could tell my problems to and who won't judge me, who won't say you are bad. I mean just imagine someone sitting there and you're getting to spill the beans, and knowing that whatever you say is going to be confidential. She's not going to criticize you. (#17)

Women who did not make a connection with the healthcare provider simply stopped seeing them. As one woman said, "Well, I tried to find other therapists, and I saw one intermittently, and I feel like it's not really that helpful, so haven't continued in that." (#11)

### Stage 2. Gaining knowledge

#### Hearing the diagnosis

Receiving a definitive diagnosis, while difficult for some women, created a sense of relief and hope, and was a turning point for the women: it signified an identifiable, manageable disorder, justified and explained how they were feeling, and created a sense of relief, comfort, and hope upon hearing that they were "not the only one" to feel this way. Many of the women had knowledge of PPD, but few knew that depression could occur during pregnancy. One woman summed up this turning point as follows:

So, it's sort of almost a relief to find out that you are not the only one, and there are actually reasons behind it. (#20)

Although many of the women were embarrassed and ashamed, they were not surprised to be diagnosed with depression; it confirmed what they "already knew". This was expressed by one participant as:

It was one of those things that you kind of know, but you don't really admit it to anyone, even to yourself. (#19)

#### Seeking information

The question that was paramount to the women was whether or not to take antidepressants. The experiences of women varied considerably in their quest for information. Some found their family physician helpful:

But, he [family physician] was very open, probably because I came with some informed sort of knowledge already, but he was very willing, he did research on it as well, and he brought a couple of studies to my attention. (#7)

Others reported that their family doctor "wasn't really equipped to deal with the question" of antidepressant use. However, all women received information about depression and its management from the psychiatrist which was "really helpful", and which many found sufficient.

She told me everything, and she also told me I could call Motherisk^1 ^[47] and ask them. I didn't call them, because I thought about it, and her information was good. (#21)

Others "wanted to check it out" for themselves and actively engaged in obtaining information via the Internet. One woman described her search for information as follows:

I started off with Motherisk and then medical journals online and abstracts. I've definitely read stuff like on Safe Parent Web Site, or Baby Centre Web Site. I tend to not trust them as much..... I don't go into the whole journals, but usually just reading the abstracts is enough to get a summary. (#7)

### Stage 3. Taking control

The process of *taking control*, which is grounded in the personal and social context of the women's lives, has three interrelated properties, making a plan, assessing progress, and balancing the risks. It is important to note that the properties making a plan, assessing *progress *and *balancing the risks *are not unidirectional, that is, the information flow and subsequent actions, may occur in either direction. As the depressive symptoms change over time, the assessment is updated, and the plan is reformulated. Nevertheless, the overall process is cyclical and moves in a clockwise direction. This is signified in the text and in Figure 1 by preceding the first word of each property with (Re).

#### (Re)Making a plan

Women spoke of "making a plan" in collaboration with their psychiatrist for managing their depression. *Making a plan *was informed by the women's recently acquired knowledge, pragmatic knowledge, and their own and their husband's values and beliefs about medication use. All women actively participated in, and took responsibility for, the management decision. Although influenced by their husband's concerns and beliefs, none of the women relinquished control of the management decisions to their husband. The act of doing so afforded the women a sense of regaining control over themselves and their lives and gave hope when they had been without hope.

It was comforting, and prepared me to say okay, how far am I willing to go, not only medication, but therapy wise. I think that set me in motion to say I am taking control over my moods, my disorder. (#16)

#### Re(Assessing) progress

Assessments of the women's mood were formally undertaken by the psychiatrist, and informally by the women themselves. If a woman's mood worsened, or if she saw herself as "stagnating", the plan was modified. Changes included increased frequency of counseling sessions, incorporation of antidepressants in a management strategy that had been free of antidepressants, or, for those already taking antidepressants, an increase in dose.

She was doing that test, the EPDS. She would check the score each time. It was very high in the beginning, and then slowly when I started taking the medication, it became low. (#21)

#### (Re)Balancing the risks

In order to determine an acceptable management plan the majority of women undertook a risk assessment of the available management options. Accepting psychotherapy or counseling was not problematic for any of the women. However, for many women, whether or not to take antidepressants was a difficult and multifaceted decision. The women lamented the fact that there were no "black and white" answers, and struggled with what they perceived to be a complex decision.

The women considered: the risk of untreated depression to the fetus and to themselves; the likelihood of developing PPD and the associated risk to the baby, themselves and their family; and the potential risks to the baby of antidepressant use during pregnancy. One woman articulated her concerns as follows:

You want to put the baby first, but, at the same time, you're just balancing out what is the risk to the baby of having a mom who is on Prozac versus what is the risk to the baby of having a mom who is, really can't cope and is falling apart. I kind of got to the point where I was like, well, I can only do the best I can as a mom. (#7)

Women tried to decrease the perceived risk to the fetus by taking as low an antidepressant dose as possible. For example, "I was kind of just teetering on, like I was trying to take the lowest dosage possible to treat my symptoms." (#18)

## Consequences of Becoming the best mom that I can – Regrounding self and regaining control

Regrounding self and regaining control describes the consequences of the strategies undertaken in confining the threat. All women had undergone counseling; ten took antidepressant medication during pregnancy, five commenced antidepressant medication within two months of delivering, and one woman delayed commencing antidepressant medication until she had weaned her baby. Three women elected not to use antidepressants at anytime during the pre- and postpartum periods. The properties of this category are: Being in a better place and Knowing self.

### Being in a better place

As a result of counseling, medication, or a combination of both, most of the women felt that they had arrived at a better place. Life still had moments of being "up and down" but, there were "far less bad days than there are good days". One woman, who managed her depression with a combination of counseling and antidepressant medication said:

I'm in a much better place now than I was before even becoming pregnant. I still have ups and downs, but my ability to deal with some of the things that are triggers for me, is much better. (#18)

Another woman, who had received counseling, but remained medication-free during the obstetric and postpartum periods, explained how she was feeling:

For me I know it's slowly going. I do get depressed sometimes. I still do get angry, exasperated. I still haven't regained my full patience. It will take work, and that's what I believe – understand, it's a work in progress. It's like building a beautiful couture dress, it takes time. It's a work of art. It takes time. You are the art piece, and you are just slowly, you know, getting primped up. (#17)

### Knowing self

All women felt that they had learned about the self and were regaining control of their life. Many felt confident that they were better able to recognize their needs, what triggered their moods, and that they could identify ways and means to ensure that those needs were met:

There's so much more to juggle. When you come home, you be [come] a wife, you be [come] a mother, and ... something, one time or another has got to give, you can't always be catching the ball. You need to take a break to recharge. I think that's important, sometimes we just forget to recharge. (#3)

Some women felt that having had depression and attended to its management, that they had undergone a "growing experience". One woman expressed this sentiment as:

I learned about myself. It was almost a gift in that, I don't know how to describe this..... I learned about myself... I've learned to take time for myself. (#19)

Some were surprised to realize that they had not been able to communicate how they had felt when they were at their lowest. As well, many identified a previous lack of awareness about the possibility of experiencing depression during pregnancy:

I never thought that I would have worries. I didn't know that I should look out for, you know, these types of things. (#16)

As the research participants recounted their experiences, many told of why they had participated in this study. Women felt it was important that other women be aware that depression can occur during pregnancy, and if it does, women should talk about their problems. Indeed, all of the women in this study identified a need to talk as part of the therapeutic process.

One of the reasons I wanted to do this study was because I really think it's important for women that are pregnant, either the first time, the second time, that if they really think that they can't handle it any more, they really need to talk to somebody. It's important. (#3)

## Discussion

This highly selected group of women with depression during pregnancy engaged fully in this research which appeared to have great salience for them. For the women in this study, becoming the best mom that I can was a complex process requiring that they recognize the problem, deal with their shame and embarrassment, identify an understanding healthcare provider, and consider the consequences of the depression and its management in order to reground the self and regain control of their lives.

There are some similarities and major differences between the findings of this study and those that have examined PPD [22] and other postpartum disorders [48] Depressive symptoms, the resulting loss of control, altered perception of self, and doubts about maternal ability described by the women in this study, appear similar to the findings generated by Beck's [22] metasynthesis of qualitative studies of PPD. The themes that emerged from her analysis, "incongruity between expectations and the reality of motherhood, spiraling downward, and pervasive loss" suggest that the experience of the depression itself may be similar for the two groups. The findings from Beck's [22] metasynthesis have drawn attention to the women's sense of loss (of control, self, relationships, and voice) as being a pervasive component of PPD. This knowledge has provided new perspectives for understanding women's experiences, [49] and prompted the use of loss and grief frameworks "in designing interventions for postpartum-depressed women" [22]

The women in the sample sought care from mental healthcare providers who were experts in the field of reproductive mental health. For some women, this was facilitated by their obstetric care providers. For others, particularly those with a pre-existing depression, seeking expert care was of their own volition. They considered that only a psychiatrist experienced in the care of pregnant and postpartum women was acceptable. This is similar to the findings of Robertson and Lyons, [48] who reported that women in their study identified themselves as needing "specialized forms of treatment" outside of that offered "within a general psychiatric service".

Finally, the women in the current study all needed to hear that "they were not the only one", in order to normalize their experiences and to gain hope for the future. For many women, the idea that depression could occur during pregnancy was antithetical to their vision of the pregnant self. Consequently, they felt embarrassed and ashamed. Goffman [50], in his essay on stigma, notes that a known discrepancy between one's expected and actual identities, "spoils" a person's "social identity", causing that person to withdraw from society and from the self (p. 31). This may explain the women's shame and inability or unwillingness to talk to friends about how they were feeling.

Having a confidante has been shown to be an important aspect of emotional support [51]. However, none of the women in this study had a confidante outside of their partner, occasionally their mother, and their mental healthcare provider. Berggren-Clive [52] noted that postpartum support groups play an "integral part of the help seeking process and important role in the creation of hope" for women with PPD. Conversely, for the women in the current study, there were no support groups. Prenatal classes currently focus on preparing the expectant mother for the birth; those classes would be an ideal venue for the provision of information and education on the possibility of mood disorders during pregnancy. Indeed, increasing depression literacy, that is, improving community awareness and understanding of depression during pregnancy, may result in the successful implementation of prevention, intervention and treatment programs for these women.

Many of the women in the current study reported stable marital relationships that strengthened after having gone through counseling. This is inconsistent with the findings of Beck [53] and Robertson & Lyons [48] who reported that the women in their studies experienced a loss of relationship with the partner. It may be that the high SES of the women in the current study protected the relationship and of partner support. Alternatively, it may be that the outcomes of depression during pregnancy and those of the postpartum differ. During pregnancy the husband may feel a particularly strong need to protect his wife and coming child. This protective role may be unsustainable by the husband when he encounters the stress and demands of a new baby and a wife who herself is not coping. This suggestion is supported by the findings of one study that has examined PPD from the perspective of the male partner [54]. Those authors report that the men's experiences of their partner's PPD was overwhelming, isolating, stigmatizing, and frustrating. Those feelings may contribute to the deterioration in the interpersonal relationships that has been reported in PPD. If this is so, appropriate and efficacious interventions for perinatal depression should include both partners.

Constructivists frequently encounter the charge of relativism: if all meanings are co-created, how can one researcher's meaning be any more important than any other meaning. However, by interpreting "*a *reality" (p.523) [24] the substantative theory developed in this study begins the process of representing the voices of the women, illuminating their perspectives and how their perspectives influence action. The theory "constitute(s) not a new truth, but a sort of tentative truth claim" about the process of managing depression during pregnancy [55]. It is context specific and thus limited in that it was developed with Canadian women. Additionally, the women in this study were all of relatively high SES, the majority in stable relationships with supportive husbands. Whether depressed obstetric patients with different characteristics would tell the same story is an area that requires further investigation. Also, it must be kept in mind that the pregnancy of interest, that is the pregnancy during which the woman was depressed, occurred on average 1.0 years (range = 0 to 2.5 years) prior to the interview. For some women this long recall time, and the events in the intervening period, may have affected their memories.

Finally, the women were recruited through a reproductive mental health program located in a tertiary hospital. Including only such women may have influenced the findings in a number of ways. First, women were in possession of knowledge, or had access to others with the knowledge, that enabled them to access such a resource. It is unlikely that the majority of obstetric patients with this disorder, especially those living within a rural setting, would have the same opportunity and therefore the same outcomes. Second, psychiatrists who see many obstetric patients may manage the disorder more confidently, that is, they may be more likely to prescribe antidepressants for pregnant women than would family physicians. Third, it is likely that there is a high degree of unrecognized and untreated depression during the obstetric period, however, the proportion of pregnant women who suffer without treatment are unknown [21]. Women who remain undiagnosed may have very different experiences than the women who participated in the current study. High quality care for women can only begin with the recognition of symptoms and an accurate diagnosis of the disorder.

Like all grounded theories, the current findings may or may not be transferable [56]. However, information has been provided so that the reader can determine whether the findings are applicable to a new situation [45]. The major strength of the grounded theory approach is that it allows for a "fuller use of highly contextualized research for ongoing discovery" [57]. Through the "continued use of emergent fit, the theory can be expanded, revised, and adjusted to maintain its usefulness" [56] in explaining the process of managing depression during pregnancy.

## Conclusion

The uniqueness of this grounded theory study is its rich description and comprehensive explanation of the strategies used by the women when managing depression during pregnancy. This project's results provide ample evidence of the need to conceptualize and manage depression during the prenatal period differently to depression during the postpartum period. Although there are similarities in the impact on functional capacity between the two periods, the challenge for a woman who is pregnant is that effective care for her may jeopardize her baby's future health. This provides a different dilemma for about-to-be parents and their healthcare providers.

An improved awareness of depression during pregnancy, on the part of health professionals, is needed to improve the women's understanding of this disorder and their ability to recognize and seek help with depression should it occur during the prenatal period. Providing information on mood disorders during pregnancy in community antenatal classes may facilitate a decrease in the shame and stigma surrounding depression during pregnancy and decrease the inadequacy felt by the women. Further qualitative research is needed to determine the specific aspects that need to be addressed in such classes.

The findings reported here contribute to an increased understanding of the challenges faced by women experiencing clinical depression during pregnancy, and identify ways in which they might be supported in confronting those challenges. Pregnancy is not always a time of blissful contentment, and those to-be mothers who have the misfortune to become clinically depressed have a difficult path to traverse. This study provides insight into the lived experiences of the journey from the viewpoint of women who have traveled that road.

## Competing interests

The authors declare that they have no competing interests.

## Authors' contributions

All authors contributed to the conceptualisation of the study, the protocol design, the overall study management, interpretation of the data, and the writing of the paper. HAB conducted the study, collected and analysed the data, and drafted the manuscript under the supervision of HSB, SR, and PG. All authors read and approved the manuscript.

## Note

^1^Motherisk is a teratogen information counseling service based in Toronto, Canada. This program offers women who are pregnant, planning, or breastfeeding, a phone-in help service to answers questions about the risk or safety of medication use.

## Pre-publication history

The pre-publication history for this paper can be accessed here:


